# Supplementary material for: Integrating Recombinase-Based Feedback and Feedforward Control for Optimal Resource Decoupling
Source: bioRxiv. 2025 May 14:2025.05.13.653855. Preprint. [Version 1] doi: 10.1101/2025.05.13.653855 (PMC12132248; doi:10.1101/2025.05.13.653855)
Supplement: Supplement 1 [file media-1.pdf]

Supplementary Information

Integrating Recombinase-Based Feedback and Feedforward Control for Optimal Resource Decoupling

Rixin Zhang[1], Rong Zhang[1], Xiao-Jun Tian[1,*]

[1] School of Biological and Health Systems Engineering, Arizona State University, Tempe, Arizona, 85281, United States.

*Correspondence: xiaojun.tian@asu.edu

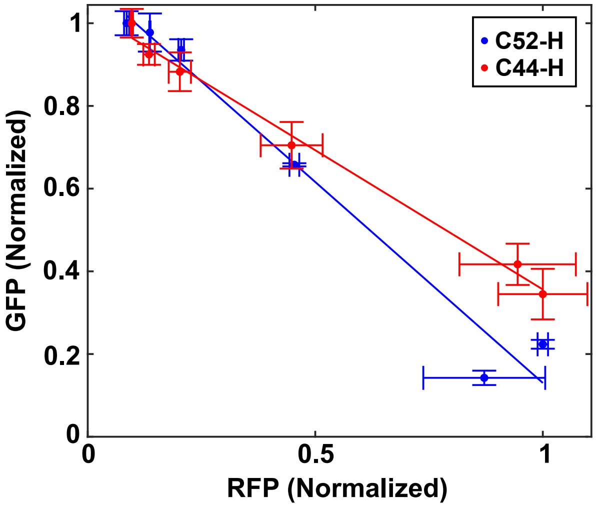

Supplementary Fig.1. Effect of high copy plasmid on the resource decoupling efficiency of Re-NF-Controller. Comparison of the correlations between GFP and RFP expression levels in circuits C44 and C52 with high plasmid copy numbers (C44-H and C52-H) across different L-ara concentrations: 0%, 1.25×10^{-4}%, 2.5×10^{-4}%, 6.25×10^{-4}%, 2.5×10^{-3}%, and 5×10^{-3}%. Solid lines represent fitted experimental data, while data points and error bars indicate mean ± s.d. (n=3).

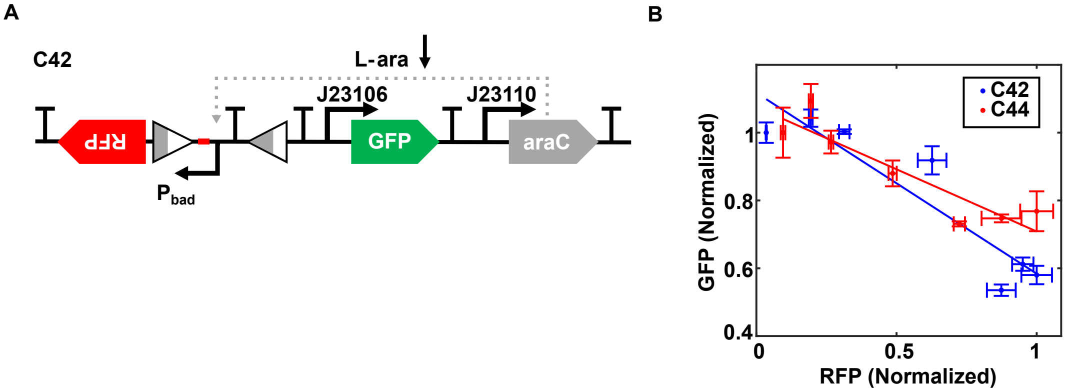

Supplementary Fig. 2. Recombinase-based negative feedback reduces resource competition in gene circuits, compared to the open-loop system without recombinase.

(A) Schematic diagram of reference circuit C42, which is an open-loop system without recombinase.

(B) Comparison of the correlations between GFP and RFP expression levels in circuits C42 and C44 across different L-ara concentrations: 0%, 1.25×10^{-4}%, 2.5×10^{-4}%, 6.25×10^{-4}%, 2.5×10^{-3}%, and 5×10^{-3}%, 8.3×10^{-3}%. Solid lines represent fitted experimental data, while data points and error bars indicate mean ± s.d. (n=3).

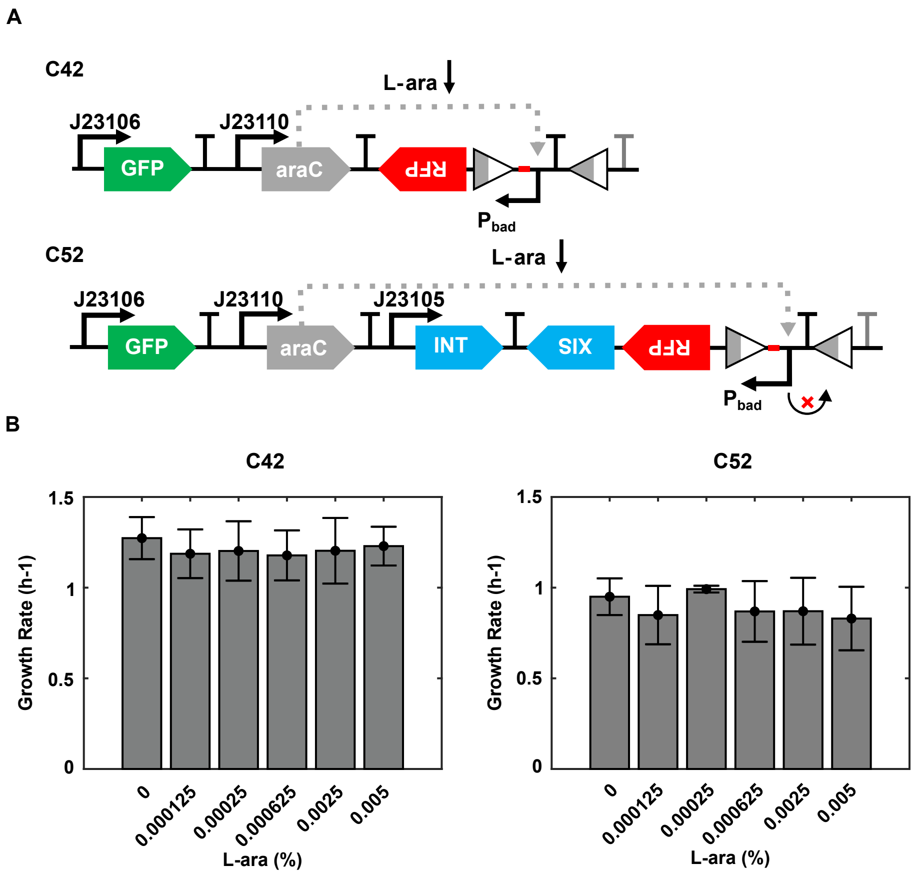

Supplementary Fig. 3. Assessment of controller gene burden as indicated by the exponential growth rate of host cells.

(A) Schematic diagram of gene circuits. The topology of C42 was rearranged to match that of C52, allowing the assessment of the burden imposed solely by the controller genes.

(B) The exponential growth rates of C42 and C52 were dynamically measured across varying L-ara concentrations: 0%, 1.25×10^{-4}%, 2.5×10^{-4}%, 6.25×10^{-4}%, 2.5×10^{-3}%, and 5×10^{-3}%. The experimental data and error bars represent means ± s.d., n=3.

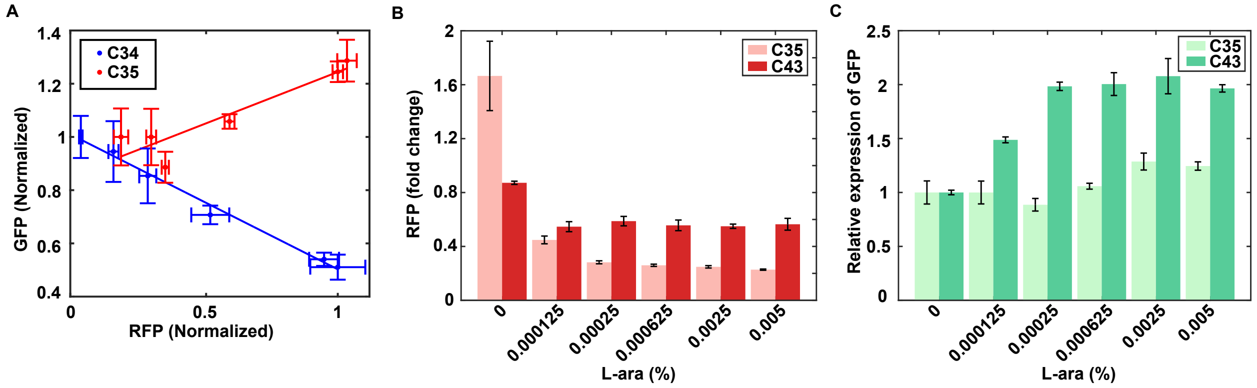

Supplementary Fig. 4. Evaluation of the impact of spacer insertion on gene expression in recombinase-modified gene circuit.

(A) The correlation between GFP and RFP expression levels in circuits C34 and C35, which are similar to C42 and C43, but with the spacer between the Pbad promoter and attL site removed. The experiments were performed across different L-ara concentrations: 0%, 1.25×10^{-4}%, 2.5×10^{-4}%, 6.25×10^{-4}%, 2.5×10^{-3}%, and 5×10^{-3}%. Solid lines represent fitted experimental data, while data points and error bars indicate mean ± s.d. (n=3).

(B) Comparison of the fold change ($\log_2$) of RFP expression between circuits without spacer (C35) and with spacer (C43), relative to their respective reference circuits C34 and C42 respectively.

(C) Comparison of the fold change of GFP expression in circuits without spacer (C35) and with spacer (C43) based on the expression in the absence of inducer.

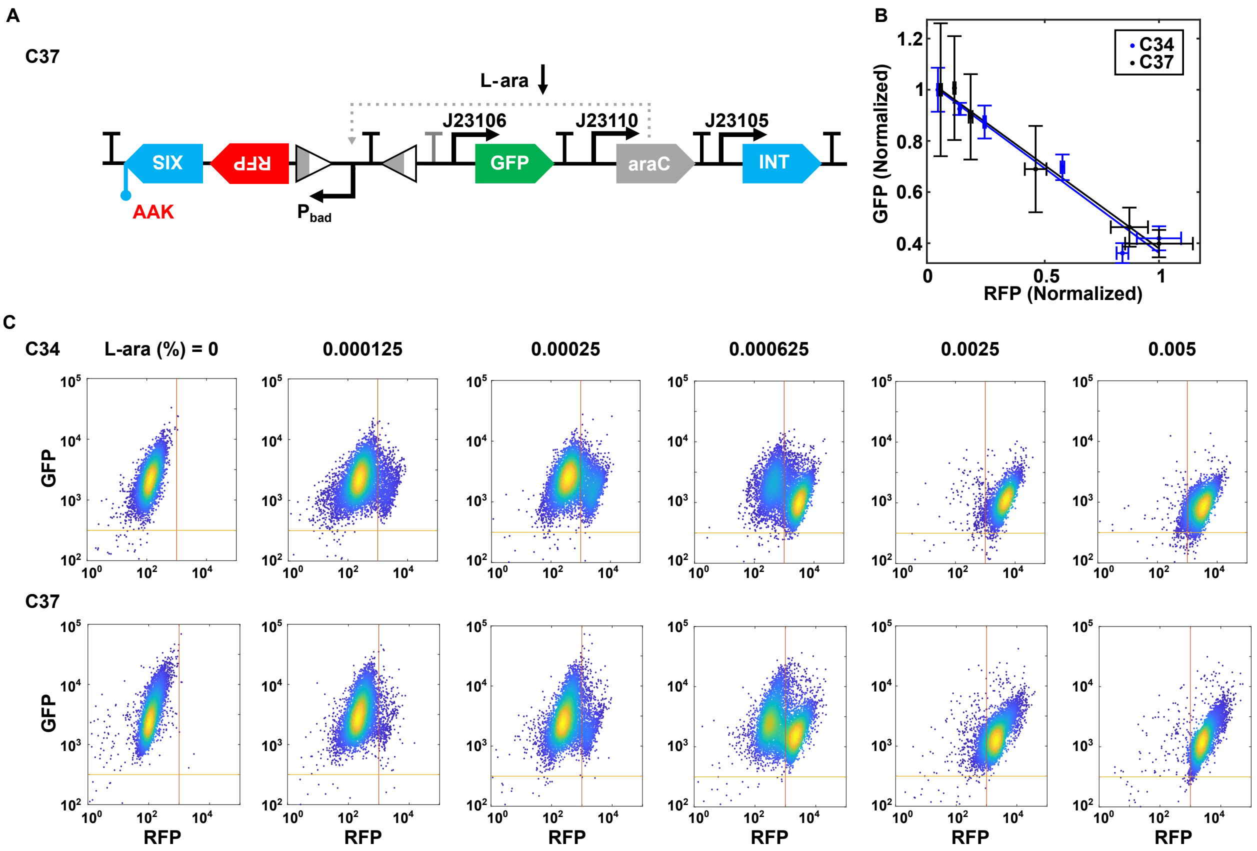

Supplementary Fig. 5. Testing the Re-NF-Controller System with a Fast Degradation Tag on Excisionase.

(A) Schematic of genetic circuit C37, where a fast degradation tag (AAK) is fused to excisionase for enhanced degradation.

(B) The correlation between GFP and RFP expression in circuit C37, showing no resource decoupling compared with the reference circuit C34. The experiments were performed across different L-ara concentrations: 0%, 1.25×10^{-4}%, 2.5×10^{-4}%, 6.25×10^{-4}%, 2.5×10^{-3}%, and 5×10^{-3}%. Solid lines represent fitted experimental data, while data points and error bars indicate mean ± s.d. (n=3).

(C) Flow cytometry data shows no change in GFP expression levels despite increased RFP expression upon L-ara induction in circuit C37, similar to the reference circuit C34. Representative results from three replicates are shown.

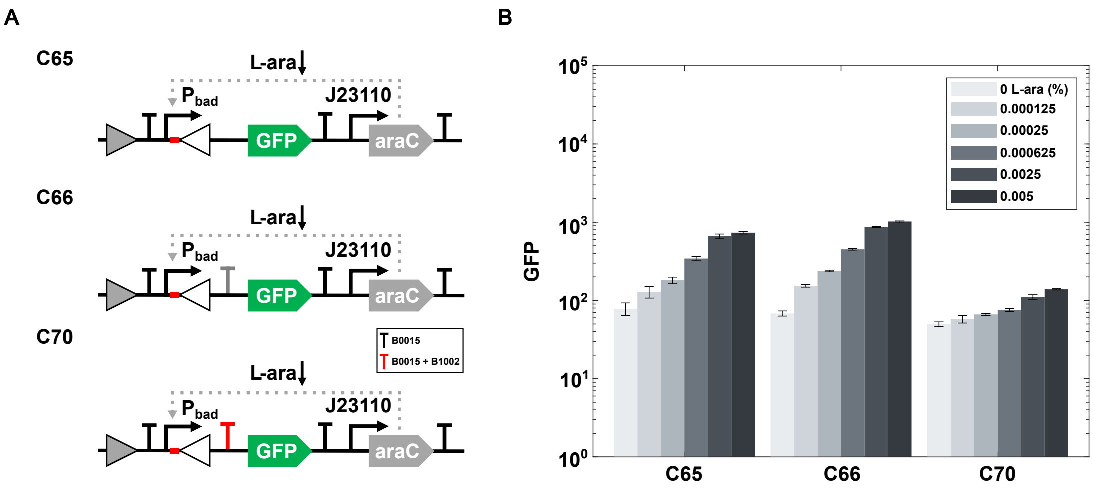

Supplementary Fig. 6. Evaluation of terminator efficiency downstream of recombinase sites after promoter flipping.

(A) Schematic diagram of genetic circuits: no terminator (C65), one terminator (B0015) (C66), or double terminators (C70) inserted downstream of Pbad.

(B) GFP expression upon induction with increasing L-ara concentrations (0%, 1.25×10^{-4}%, 2.5×10^{-4}%, 6.25×10^{-4}%, 2.5×10^{-3}%, and 5×10^{-3}%) measured using a plate reader. The experimental data and error bars represent means ± s.d., n=3.

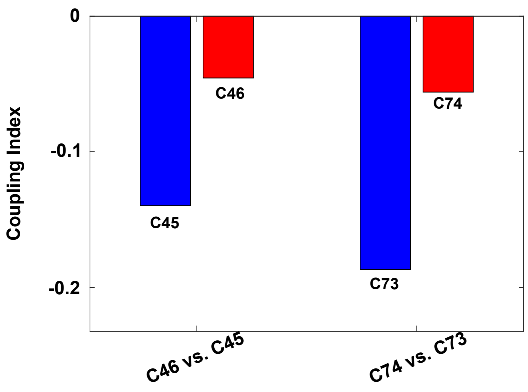

Supplementary Fig. 7. Coupling index shows the resource decoupling effect of the circuits C46 and C74 compared to their reference circuits.

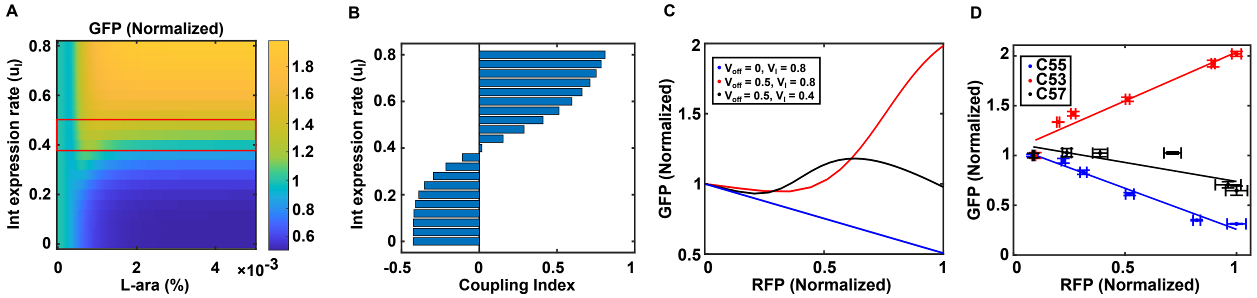

Supplementary Fig. 8. Optimizing resource competition mitigation in the Re-NF-FF-Controller by tuning the integrase transcriptional,

(A) Simulated steady-state GFP expression levels as a function of inducer concentration and expression rate of integrase. The boxed region highlights the effective resource decoupling achieved by the Re-NF-FF-Controller.

(B) The coupling index between GFP and RFP as a function of the expression rate of integrase.

(C) The optimal resource decoupling effect achieved by tuning the expression rate of integrase (black line) to correct the overcompensation (red line). The open-loop system (blue line) is shown as a reference.

(D) Correlations between GFP and RFP expression levels in circuits C53, C55, and C57 across different L-ara concentrations: 0%, 1.25×10^{-4}%, 2.5×10^{-4}%, 6.25×10^{-4}%, 2.5×10^{-3}%, and 5×10^{-3}%. Solid lines represent fitted experimental data, while data points and error bars indicate mean ± s.d. (n=3).

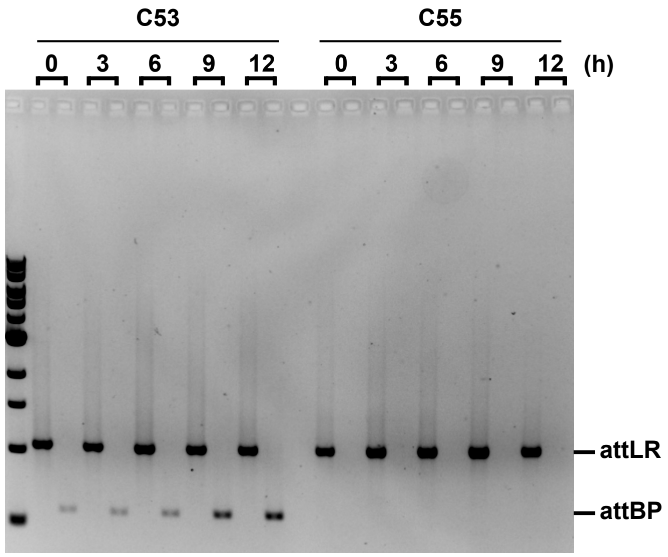

Supplementary Fig. 9. Gel electrophoresis analysis of PCR products to determine the timing of promoter flipping in circuits C53 and C55. The system with circuit C53 or C55 was induced with 5 × 10^{-3}% L-ara at different time series: 0, 3, 6, 9, 12h.

Mathematical modeling

To simulate the effect of recombinase-based control system on controlling resource competition in cellular environment, we built a mathematical model utilizing a system of ordinary differential equations (ODE).

1. Mathematical Modeling of Resource Competition in the System

Here, we present the general mathematical model for resource competition in a system, which will be used to describe and analyze system dynamics both in the absence of a controller and with the Re-NF-Controller or Re-NF-FF-Controller in subsequent sections. The dynamics of protein concentration $[P_i]$ in the system are modeled as follows:

$$\frac{d[P_i]}{dt} = v_i * \frac{CN_i * R_i/Q_i}{PF_Q} - d_i * [P_i]$$

Where CN_i is gene copy number, v_i is the expression rate of protein P_i, and d_i is the degradation rate of protein P_i. R_i is the fraction of active promoters for expressing protein P_i. For constitutive promoters, $R_\mathrm{i} = 1$. For inducible promoters, such as Pbad in our system, R_i depends on the concentration of the transcription factor, following a Hill function. For example, consider the protein controlled by Pbad promoter, $\mathrm{R_i} = \frac{Sa*[AraC]^2}{Sa*[AraC]^2+1}$, where $Sa = C_{min} + (C_{max} - C_{min}) * \left(\frac{Lara^n}{Lara^n+J^n}\right)$. Sa describes how the promoter activity is regulated by inducer L-ara, Cmax and Cmin are the maximum and minimum affinities of the AraC dimers to the binding sites on the promoter Pbad. The details about model construction for AraC activated system are further explained in our previous work (1).

It is noted that the effect of the resource competition is formulated with $\mathrm{PF_Q} = 1 + CN \sum \frac{R_\mathrm{i}}{Q_\mathrm{i}}$, where Q_i is introduced to represent the difference in resource competitivity between genes. The derivation of the equations describing resource competition can be found in our previous work (2). Building on this modeling framework described, we developed models for three systems: the open-loop system, the system with the Re-NF-Controller, and the system with the Re-NF-FF-Controller.

2. Mathematical Model for the Open-Loop System.

In the Open-Loop System, promoter flipping does not occur. We applied the general resource competition model to all proteins in the system, leading to the following set of equations:

$$\frac{d[INT]}{dt} = u_I * \frac{CN/Q_I}{PF_Q} - d_I * [INT]$$

$$\frac{d[XIS]}{dt} = u_R * \frac{CN * R_R/Q_R}{PF_Q} - d_X * [XIS]$$
$$\frac{d[AraC]}{dt} = u_A * \frac{CN/Q_A}{PF_Q} - d_A * [AraC]$$
$$\frac{d[GFP]}{dt} = u_{G1} * \frac{CN/Q_G}{PF_Q} - d_G * [GFP]$$
$$\frac{d[RFP]}{dt} = u_R * \frac{CN * R_R/Q_R}{PF_Q} - d_R * [RFP]$$

Where $PF_Q = 1 + CN * \left(\frac{1}{QI} + \frac{1}{Q_A} + \frac{1}{Q_G} + \frac{2*R_R}{Q_R}\right), R_R = \frac{Sa*[AraC]^2}{Sa*[AraC]^2+1}, Sa = C_{min} + (C_{max} - C_{min}) * \left(\frac{Lara^n}{Lara^n + J^n}\right)$.

3. Mathematical Model for the System with Re-NF-Controller

In the system with a Re-NF-Controller, the orientation of the Pbad promoter can be flipped by recombinase. Initially, all copies of the Pbad promoter are designed in the LR state to drive RFP expression. Upon induction, RFP and excisionase accumulate in the system, allowing excisionase to form a complex with the constitutively expressed integrase, which flips the Pbad promoter into the BP state. We introduce an equation to model the proportion of Pbad promoter copies remaining in the LR state $\phi \in [0,1]$

$$\frac{d[\phi]}{dt} = v_{\text{on}} * \frac{[INT]^4}{{K_{I1}}^4 + [INT]^4} * (1 - \phi) - v_{\text{off}} * \frac{[XIS]^4}{{K_X}^4 + [XIS]^4} \frac{[INT]^4}{{K_{I2}}^4 + [INT]^4} * \phi$$

where the first and second terms represent the promoter flipping rate between LR and BP states, with maximum rate constant of v_{on} and v_{off}, respectively. $K_{I1,2}$ and K_X are equilibrium constants that determine the integrase and excisionase concentration required to reach half-maximal flipping rate (3, 4). Since four integrase molecules are required for promoter flipping, the integrase-dependent fraction follows a fourth-order Hill function. The integrase-excisionase complex is assumed to have a 1:1 stoichiometry for catalyzing LR-to-BP recombination, leading to an excisionase-dependent fraction modeled by a Hill function with a coefficient of 4.

The equation for RFP needs to be revised accordingly

$$\frac{d[RFP]}{dt} = u_R * \frac{CN * \phi * R_R/Q_R}{PF_Q} - d_R * [RFP]$$

To simulate how the strength of the negative feedback loop influences resource decoupling efficiency, we systematically analyzed the system across a wide range of maximal flipping rates from the LR state to the BP state, with $v_{\text{off}} \in [0, 0.75]$.

4. Mathematical Model for the System with Re-NF-FF-Controller

In the system with a Re-NF-FF-Controller, the flipped Pbad promoter is designed to promote GFP expression. The ODE of GFP needs to be revised as follows:

$$\frac{d[GFP]}{dt} = u_{G1} * \frac{CN/Q_G}{PF_Q} + v_{G2} * \frac{(1-\phi) * CN * R_R/Q_R}{PF_Q} - d_G * [GFP]$$

The second term represents an additional expression from the flipped Pbad promoter. To simulate the tunability of the Re-NF-FF-Controller, we systematically analyzed the system across a wide range of degradation rate of excisionase ($d_X \in [0.01, 0.06]$) and the expression rate of integrase ($v_I \in [0, 0.8]$).

5. The general Mathematical model for all the systems

In this section, we unify all the above ODE models into a generalized framework. In the Open-Loop System, the Pbad promoter for the RFP module remains unchanged, meaning ϕ is fixed at 1, which can be enforced by setting v_{off} = 0. To standardize the GFP expression equation across systems with different control mechanisms, we introduce a hyperparameter λ into the second production term of GFP, allowing this term to be zero ($\lambda = 0$) for the system with only negative feedback regulation and active ($\lambda = 1$) for the system incorporating additional feedforward regulation. This allows us to formulate a single generalized model applicable to all systems,

$$\frac{d[INT]}{dt} = u_I * \frac{CN/Q_I}{PF_Q} - d_I * [INT]$$

$$\frac{d[XIS]}{dt} = u_R * \frac{CN * R_R/Q_R}{PF_Q} - d_X * [XIS]$$

$$\frac{d[AraC]}{dt} = u_A * \frac{CN/Q_A}{PF_Q} - d_A * [AraC]$$

$$\frac{d[GFP]}{dt} = u_{G1} * \frac{CN/Q_G}{PF_Q} + \lambda * u_{G2} * \frac{(1-\phi) * CN * R_R/Q_R}{PF_Q} - d_G * [GFP]$$

$$\frac{d[RFP]}{dt} = u_R * \frac{CN * \phi * R_R/Q_R}{PF_Q} - d_R * [RFP]$$

$$\frac{d[\phi]}{dt} = v_{\text{on}} * \frac{[INT]^4}{{K_{I1}}^4 + [INT]^4} * (1-\phi) - v_{\text{off}} * \frac{[XIS]^4}{{K_X}^4 + [XIS]^4} \frac{[INT]^4}{{K_{I2}}^4 + [INT]^4} * \phi$$

Where $PF_Q = 1 + CN * \left(\frac{1}{QI} + \frac{1}{Q_A} + \frac{1}{Q_G} + \frac{2*\phi*R_R}{Q_R} + \frac{(1-\phi)*R_R}{Q_R}\right), R_R = \frac{Sa*[AraC]^2}{Sa*[AraC]^2+1}, Sa = C_{min} + (C_{max} - C_{min}) * \left(\frac{Lara^n}{Lara^n+J^n}\right)$.

The parameters are, unless otherwise mentioned:

$CN = 50, v_{\text{on}} = 0.1, v_{\text{off}} = 0.5, v_A = 0.5, v_I = 0.8, v_{G1} = 0.8, v_{G2} = 2, v_R = 0.5, K_{I1} = 0.1, K_{I2} = 5, K_X = 5, J = 0.001, C_{min} = 0.000001, C_{max} = 0.015, \text{n} = 3, d_A = 0.01, d_I = 0.01, d_G = 0.01, d_R = 0.01, d_X = 0.01, Q_A = 150, Q_I = 150, Q_G = 50, Q_R = 5.$

Supplementary Table 1. BioBrick parts used in this paper.

Part name	Description	Biobrick number
J23105	Constitutive promoter	BBa_J23105
J23106	Constitutive promoter	BBa_J23106
J23110	Constitutive promoter	BBa_J23110
Ptet	Constitutive promoter	BBa_R0040
Pbad	Inducible promoter activated by AraC and L-arabinose	BBa_K206000
B0031	Ribosome binding site	BBa_B0031
B0034	Ribosome binding site	BBa_B0034
AraC	Arabinose operon regulatory protein	araC
GFP	Green fluorescent protein	BBa_E0040
RFP	Red fluorescent protein	BBa_E1010
CFP	Cyan fluorescent protein	BBa_E0020
B0015	Terminator	BBa_B0015
B1002	Terminator	BBa_B1002
PSB1A2	Plasmid Backbone	pSB1A2
PSB3K3	Plasmid Backbone	pSB3K3

Supplementary Table 2. Sequences of parts used in this paper.

Part name	Description	Sequence
INT	Integrase	atgagagccctggtagtcatccgcctgtcccgcgtcaccgatg<br>ctacgacttcaccggagcgtcagctggagtcttgccagcagct<br>ctgcgcccagcgcggctgggacgtcgtcggggtagcggagg<br>atctggacgtctccggggcggtcgatccgttcgaccggaagc<br>gcagaccgaacctggcccggtggctagcgttcgaggagcaa<br>ccgtttgacgtgatcgtggcgtaccgggtagatcggttgacccg<br>atcgatccggcatcttcagcagctggtccactgggccgaggac<br>cacaagaagctggtcgtctccgcgaccgaagcgcacttcgat<br>acgacgacgccgtttgcggcggtcgtcatcgcgcttatgggaa<br>cggtggcgcagatggaattagaagcgatcaaagagcggaa<br>ccgttcggctgcgcatttcaatatccgcgccgggaaataccga<br>ggatccctgccgccgtggggatacctgcctacgcgcgtggac<br>ggggagtggcggctggtgccggaccctgtgcagcgagagcg<br>catcctcgaggtgtatcaccgcgtcgtcgacaaccacgagcc<br>gctgcatctggtggcccacgacctgaaccggcgtggtgtcctgt<br>cgccgaaggactacttcgcgcagctgcaaggccgcgagccg<br>cagggccgggagtggtcggctaccgcgctgaagcgatcgat<br>gatctccgaggcgatgctcgggtacgcgactctgaacggtaa<br>gaccgtccgagacgacgacggagccccgctggtgcgggctg<br>agccgatcctgacccgtgagcagctggaggcgctgcgcgcc<br>gagctcgtgaagacctcccgggcgaagcccgcggtgtctacc<br>ccgtcgctgctgctgcgggtgttgttctgcgcggtgtgcggga<br>gcccgcgtacaagttcgccgggggaggacgtaagcacccgc<br>gctaccgctgccgctcgatggggttcccgaagcactgcgga<br>acggcacggtggcgatggccgagtgggacgcgttctgcgag<br>gagcaggtactggatctgctcggggacgcggagcgtctggag<br>aaagtctgggtagcgggctcggactccgcggtcgaactcgcg<br>gaggtgaacgcggagctggtggacctgacgtcgctgatcggc<br>tccccggcctaccgggcgggctctccgcagcgagaagcact<br>ggatgcccgtattgcggcgctggccgcgcggcaagaggagc<br>tggagggcctggaggctcgcccgtctggctgggagtggcgcg<br>agaccgggcagcggttcggggactggtggcgggagcagga<br>caccgcggcaaagaacacctggcttcggtcgatgaacgttcg<br>gctgacgttcgacgtccgcggcgggctgactcgcacgatcga<br>cttcggggatcttcaggagtacgagcagcatctcaggctcggc<br>agcgtggtcgaacggctacacaccgggatgtcgtaa(5)
XIS	Excisionase	atggccggcactcagcgtatcgtctttctacccgatactcagttg<br>cctttcgaggcgcgcaaagagatgcaagcggtcatccgcttc<br>atcggggatgtccagccgtacggcgtggtacatatcggtgacg<br>tcctagacctgccgcagccctcgcgctggaaccggggaacc<br>aagggcgagttcgagggctcggtgtaccgcgacgcggacta<br>cgccaagaagaacctgatggagccgctgcgcaaggtctacg<br>acggctggatcgggatgcacgagggcaaccacgatctgcga<br>gcccgcgagtacctggccaagaacgcaccggccctggagg<br>gtacgcacgctttcgacatcgacgtgctgctcgacttcgacggg<br>ttcggtgtggagctgctgcctgacttctacgacatcgctccgggc<br>tggatctccactcacgggcacatgggcaagatgacgctatccc<br>agatcgccggatcgacagcgctcaacggtgccaagaagttc

		ggcaagtccgtggtctgcggccacacgcaccggcaggctgtc gtctcgcactcgttcgggtacggcggctcggtgcgcaagaccg tcaccggcatggaagtcgggcacctgatggacatgaagaag gccaactatctaaagggcggagccgggaactggcagatggg cttcgggatgctcacggtcgacggcaagcatgtcaaggctga gatcgtcccgatcctgggaggcaagttcaccgttgacggcca ggtctgggaagtcaccggt(5)
attB	Attachment site attB	cggccggcttgtcgacgacggcgcactccgtcgtcaggatcat ccgggc(6)
attP	Attachment site attP	gtcgtggtttgtctggtcaaccaccgcgcactcagtggtgtacg gtacaaaccccgac(6)
attL	Attachment site attL	cggccggcttgtcgacgacggcgcactcagtggtgtacggta caaaccccgac
attR	Attachment site attR	gtcgtggtttgtctggtcaaccaccgcgcactccgtcgtcaggat catccgggc
attR-M	Mutated attachment site attR	gtcgtggtttgtctggtcaaccaccgcggtctccgtcgtcaggat catccgggc
AAK-tag	Fast degradation tag	gcagcaaacgacgaaaactacgctgctgctaaa
LVA-tag	Fast degradation tag	gctgcaaacgacgaaaactacgctttagtagct
Spacer	Spacer	agatggttagtgtgcgtatctgatacttttatgtctgtcctacaactc caagcgtacagttcaggccaacaccctagctttgagaaagtc gcgtcagtaaatgtcgtttccctaaagcttacaatttacctcctcg ctatt

Supplementary Table 3. List of circuit assembly.

Circuit name	Circuit design*
C5	J23110 + B0034 + AraC + B0015 + B0015 (R) + RFP-Lva (R) + B0034 (R) + attB + B0015 (R) + Pbad + attp (R) + B0034 + GFP-Lva + B0015 + PSB1A2
C6	J23110 + B0034 + AraC + B0015 + B0015 (R) + RFP-Lva (R) + B0034 (R) + attB + B0015 (R) + Pbad + attp (R) + B0034 + INT + B0034 + GFP-Lva + B0015 + PSB1A2
C21	J23110 + B0034 + AraC + B0015 + B1002 (R) + XIS (R) + B0034 (R) + RFP (R) + B0034 (R) + attL + Pbad (R) + B0015 + attR (R) + B0034 + GFP + B0015 + PSB1A2
C24	J23110 + B0034 + AraC + B0015 + B1002 (R) + XIS (R) + B0034 (R) + RFP (R) + B0034 (R) + attL + Pbad (R) + B0015 + attR (R) + B0034 + GFP + B0015 + J23105 + B0031 + INT + B0015 + PSB1A2
C25	J23110 + B0034 + AraC + B0015 + B1002 (R) + XIS (R) + B0034 (R) + RFP (R) + B0034 (R) + attL + Pbad (R) + B0015 + attR (R) + B0034 + GFP + B0015 + J23105 + B0031 + INT (start codon ATG to GTG) + B0015 + PSB1A2
C34	B1002 (R) + RFP (R) + B0034 (R) + attL + Pbad (R) + B0015 + attR (R) + B0015 + J23106 + B0034 + GFP + B0015 + J23110 + B0034 + AraC + B0015 + PSB3K3
C35	B1002 (R) + XIS (R) + B0034 (R) + RFP (R) + B0034 (R) + attL + Pbad (R) + B0015 + attR (R) + B0015 + J23106 + B0034 + GFP + B0015 + J23110 + B0034 + AraC + B0015 + J23105 + B0031 + INT + B0015 + PSB3K3
C37	B1002 (R) + AAK-tag (R) + XIS (R) + B0034 (R) + RFP (R) + B0034 (R) + attL + Pbad (R) + B0015 + attR (R) + B0015 + J23106 + B0034 + GFP + B0015 + J23110 + B0034 + AraC + B0015 + J23105 + B0031 + INT + B0015 + PSB3K3
C42	B1002 (R) + RFP (R) + B0034 (R) + attL + Spacer (R) + Pbad (R) + B0015 + attR (R) + B0015 + J23106 + B0034 + GFP + B0015 + J23110 + B0034 + AraC + B0015 + PSB3K3
C43	B1002 (R) + XIS (R) + B0034 (R) + RFP (R) + B0034 (R) + attL + Spacer (R) + Pbad (R) + B0015 + attR (R) + B0015 + J23106 + B0034 + GFP + B0015 + J23110 + B0034 + AraC + B0015 + J23105 + B0031 + INT + B0015 + PSB3K3
C44	J23106 + B0034 + GFP + B0015 + J23110 + B0034 + AraC + B0015 + J23105 + B0031 + INT + B0015 + B1002 (R) + XIS (R) + B0034 (R) + RFP (R) + B0034 (R) + attL + Spacer (R) + Pbad (R) + B0015 + attR (R) + B0015 + PSB3K3
C45	B1002 (R) + RFP (R) + B0034 (R) + attL + Spacer (R) + Pbad (R) + B0015 + attR (R) + B0015 + J23106 + B0034 + GFP + B0015 + J23110 + B0034 + AraC + B0015 + Ptet + B0034 + CFP + B0015 + PSB3K3
C46	B1002 (R) + XIS (R) + B0034 (R) + RFP (R) + B0034 (R) + attL + Spacer (R) + Pbad (R) + B0015 + attR (R) + B0015 + J23106 + B0034 + GFP + B0015 + J23110 + B0034 + AraC + B0015 + J23105 + B0031 + INT + B0015 + Ptet + B0034 + CFP + B0015 + PSB3K3
C52	J23106 + B0034 + GFP + B0015 + J23110 + B0034 + AraC + B0015 + J23105 + B0031 + INT + B0015 + B1002 (R) + XIS (R) + B0034 (R) + RFP (R) + B0034 (R) + attL + Spacer (R) + Pbad (R) + B0015 + attR-M (R) + B0015 + PSB3K3

C53	B1002 (R) + XIS (R) + B0034 (R) + RFP (R) + B0034 (R) + attL + Spacer (R) + Pbad (R) + B0015 + attR (R) + J23106 + B0034 + GFP + B0015 + J23110 + B0034 + AraC + B0015 + J23105 + B0031 + INT + B0015 + PSB3K3
C55	B1002 (R) + XIS (R) + B0034 (R) + RFP (R) + B0034 (R) + attL + Spacer (R) + Pbad (R) + B0015 + attR-M (R) + J23106 + B0034 + GFP + B0015 + J23110 + B0034 + AraC + B0015 + J23105 + B0031 + INT + B0015 + PSB3K3
C57	B1002 (R) + XIS (R) + B0034 (R) + RFP (R) + B0034 (R) + attL + Spacer (R) + Pbad (R) + B0015 + attR (R) + J23106 + B0034 + GFP + B0015 + J23110 + B0034 + AraC + B0015 + J23105 + B0031 + INT (start codon ATG to GTG) + B0015 + PSB3K3
C59	B1002 (R) + AAK-tag (R) + XIS (R) + B0034 (R) + RFP (R) + B0034 (R) + attL + Spacer (R) + Pbad (R) + B0015 + attR (R) + J23106 + B0034 + GFP + B0015 + J23110 + B0034 + AraC + B0015 + J23105 + B0031 + INT + B0015 + PSB3K3
C65	attB + B0015 (R) + Pbad + Spacer + attP (R) + B0034 + GFP + B0015 + J23110 + B0034 + AraC + B0015 + PSB3K3
C66	attB + B0015 (R) + Pbad + Spacer + attP (R) + B0015 + B0034 + GFP + B0015 + J23110 + B0034 + AraC + B0015 + PSB3K3
C70	attB + B0015 (R) + Pbad + Spacer + attP (R) + B0015 + B1002 + B0034 + GFP + B0015 + J23110 + B0034 + AraC + B0015 + PSB3K3
C73	B1002 (R) + RFP (R) + B0034 (R) + attL + Spacer (R) + Pbad (R) + B0015 + attR (R) + B0015 + B1002 + J23106 + B0034 + GFP + B0015 + J23110 + B0034 + AraC + B0015 + PSB3K3
C74	B1002 (R) + XIS (R) + B0034 (R) + RFP (R) + B0034 (R) + attL + Spacer (R) + Pbad (R) + B0015 + attR (R) + B0015 + B1002 + J23106 + B0034 + GFP + B0015 + J23110 + B0034 + AraC + B0015 + J23105 + B0031 + INT + B0015 + PSB3K3

* (R) indicates that the gene is arranged in reverse orientation within the circuit.

Reference:

1. Zhang,R., Li,J., Melendez-Alvarez,J., Chen,X., Sochor,P., Goetz,H., Zhang,Q., Ding,T., Wang,X. and Tian,X.-J. (2020) Topology-dependent interference of synthetic gene circuit function by growth feedback. *Nat Chem Biol*, **16**, 695–701.
2. Zhang,R., Goetz,H., Melendez-Alvarez,J., Li,J., Ding,T., Wang,X. and Tian,X.-J. (2021) Winner-takes-all resource competition redirects cascading cell fate transitions. *Nat Commun*, **12**, 853.
3. Folliard,T., Steel,H., Prescott,T.P., Wadhams,G., Rothschild,L.J. and Papachristodoulou,A. (2017) A Synthetic Recombinase-Based Feedback Loop Results in Robust Expression. *ACS Synth Biol*, **6**, 1663–1671.
4. Steel,H. and Papachristodoulou,A. (2019) Low-Burden Biological Feedback Controllers for Near-Perfect Adaptation. *ACS Synth Biol*, **8**, 2212–2219.
5. Bonnet,J., Subsoontorn,P. and Endy,D. (2012) Rewritable digital data storage in live cells via engineered control of recombination directionality. *Proceedings of the National Academy of Sciences*, **109**, 8884–8889.
6. Roquet,N., Soleimany,A.P., Ferris,A.C., Aaronson,S. and Lu,T.K. (2016) Synthetic recombinase-based state machines in living cells. *Science (1979)*, **353**, aad8559.
